# Lysyl oxidases regulate fibrillar collagen remodelling in idiopathic pulmonary fibrosis

**DOI:** 10.1242/dmm.030114

**Published:** 2017-11-01

**Authors:** Gavin Tjin, Eric S. White, Alen Faiz, Delphine Sicard, Daniel J. Tschumperlin, Annabelle Mahar, Eleanor P. W. Kable, Janette K. Burgess

**Affiliations:** 1 Respiratory Cellular and Molecular Biology Group, Woolcock Institute of Medical Research, Glebe, New South Wales 2037, Australia; 2 Central Clinical School, Faculty of Medicine, The University of Sydney, Sydney, New South Wales 2006, Australia; 3 Australian Centre for Microscopy and Microanalysis, The University of Sydney, Sydney, New South Wales 2006, Australia; 4 Stem Cell Regulation Unit, St. Vincent's Institute of Medical Research, Victoria 3065, Australia; 5 Pulmonary & Critical Care Medicine, Department of Internal Medicine, University of Michigan, Ann Arbor, MI 48109, USA; 6 University of Groningen, University Medical Center Groningen, Department of Pulmonary Diseases, Groningen, 9713 GZ, The Netherlands; 7 University of Groningen, University Medical Center Groningen, Department of Pathology & Medical Biology, Experimental Pulmonology and Inflammation Research, Groningen, 9713 GZ, The Netherlands; 8 University of Groningen, University Medical Center Groningen, GRIAC (Groningen Research Institute for Asthma and COPD), Groningen, 9713 GZ, The Netherlands; 9 Department of Physiology & Biomedical Engineering, College of Medicine, Mayo Clinic, Rochester, MN 55905, USA; 10 Department of Tissue Pathology and Diagnostic Oncology, Royal Prince Alfred Hospital, Sydney, New South Wales 2050, Australia; 11 Discipline of Pharmacology, The University of Sydney, Sydney, New South Wales 2006, Australia

**Keywords:** Idiopathic pulmonary fibrosis, Extracellular matrix, Collagen, Lysyl oxidase, Second harmonic generation

## Abstract

Idiopathic pulmonary fibrosis (IPF) is a progressive scarring disease of the lung with few effective therapeutic options. Structural remodelling of the extracellular matrix [i.e. collagen cross-linking mediated by the lysyl oxidase (LO) family of enzymes (LOX, LOXL1-4)] might contribute to disease pathogenesis and represent a therapeutic target. This study aimed to further our understanding of the mechanisms by which LO inhibitors might improve lung fibrosis. Lung tissues from IPF and non-IPF subjects were examined for collagen structure (second harmonic generation imaging) and LO gene (microarray analysis) and protein (immunohistochemistry and western blotting) levels. Functional effects (collagen structure and tissue stiffness using atomic force microscopy) of LO inhibitors on collagen remodelling were examined in two models, collagen hydrogels and decellularized human lung matrices. *LOXL1*/*LOXL2* gene expression and protein levels were increased in IPF versus non-IPF. Increased collagen fibril thickness in IPF versus non-IPF lung tissues correlated with increased LOXL1/LOXL2, and decreased LOX, protein expression. β-Aminoproprionitrile (β-APN; pan-LO inhibitor) but not Compound A (LOXL2-specific inhibitor) interfered with transforming growth factor-β-induced collagen remodelling in both models. The β-APN treatment group was tested further, and β-APN was found to interfere with stiffening in the decellularized matrix model. LOXL1 activity might drive collagen remodelling in IPF lungs. The interrelationship between collagen structural remodelling and LOs is disrupted in IPF lungs. Inhibition of LO activity alleviates fibrosis by limiting fibrillar collagen cross-linking, thereby potentially impeding the formation of a pathological microenvironment in IPF.

## Abstract (precis)

**Summary:** Transforming growth factor-β-induced collagen remodelling is driven by enhanced lysyl oxidase expression in idiopathic pulmonary fibrosis.

## INTRODUCTION

Idiopathic pulmonary fibrosis (IPF) is a specific form of progressive fibrosing lung disease of unknown cause that leads to chronic respiratory failure and death (Raghu et al., 2011), with a median survival of ∼3 years after diagnosis (Demedts et al., 2001). IPF is a subcategory under the general disease category of interstitial lung disease (ILD). Common traits of ILDs are the presence of inflammation and pulmonary fibrosis. IPF patients have a poor prognosis, with 44% mortality rate for IPF patients expected within 5 years, compared with the mortality rates in two other ILDs, ILD-associated connective tissue disease (33%) and pulmonary sarcoidosis (2%) (Demedts et al., 2001).

Like many fibrotic disorders, IPF is characterized by enhanced deposition and remodelling of the extracellular matrix (ECM). Although data are emerging about the altered composition of the ECM in IPF (Booth et al., 2012; Estany et al., 2014; Kuhn et al., 1989; Kuhn and McDonald, 1991; Ward and Hunninghake, 1998), less is known about the structural changes that occur in collagens in this disease. Collagens undergo extensive post-translational modifications, including formation of cross-links between fibres that stabilize the molecules and generate a strong collagen fibril, which provides tensile strength. Aberrant cross-linking is known to contribute to increased tissue stiffness, a pathological characteristic of IPF (Clarke et al., 2013).

Fibrillar collagen structures (predominantly collagens I and III) can be visualized and quantified using second harmonic generation (SHG) microscopy (Abraham et al., 2012; Tjin et al., 2014). SHG imaging has been used to analyse ECM structures in biological systems *in vitro*, *ex vivo* and *in vivo* (Brown et al., 2003; Caetano-Lopes et al., 2010; Cox et al., 2003; Guo et al., 1999; Nadiarnykh et al., 2007; Sivaguru et al., 2010; Suzuki et al., 2012; Williams et al., 2001) and can semi-quantitatively measure the ratio of mature organized collagen to immature disorganized collagen fibrils (Williams et al., 2005). This technique, therefore, enables comparisons of collagen rearrangement in tissues, analysis of collagen structure (Caetano-Lopes et al., 2010; Nadiarnykh et al., 2007; Sivaguru et al., 2010; Suzuki et al., 2012) and even measurement of the thickness of the fibres (Chu et al., 2007). We have recently reported the robustness of this technique to quantify fibrillar collagen I remodelling in airway tissues in chronic obstructive pulmonary disease (Tjin et al., 2014).

Maturity of collagen fibres and thus the mechanical properties of the fibres are dependent on the level of intermolecular cross-linking. Although the intermolecular fibrillar organization and certain cross-linking processes can happen spontaneously, enzymes such as lysyl oxidases (LOs) are necessary for further cross-linking. The LO family of enzymes consists of five known paralogues: lysyl oxidase (LOX) and LOX like 1-4 (LOXL1-4). During normal development, the expression of LOs is tightly regulated, with aberrant expression and activity being associated with pathological manifestation in various diseases, including cancer (Erler et al., 2006; Payne et al., 2005) and, as recently discovered, IPF (Aumiller et al., 2017; Barry-Hamilton et al., 2010).

Collagen cross-linking contributes to the stiffness of the ECM of tissues and is tightly regulated in normal tissue homeostasis. However, in diseased tissue, aberrant cross-linking could lead to the development of a pathological microenvironment. The stiffness of the ECM influences the behaviour and function of cells within its vicinity, including an increase in fibroblast proliferation and contraction (Balestrini et al., 2012; Marinkovic et al., 2013). In addition, a stiffer ECM leads to an increase in latent transforming growth factor-β (TGF-β) activation (Burgess et al., 2016; Shi et al., 2011; Wipff et al., 2007). In IPF, the lung tissue has a stiffness of 16.52±2.25 kPa, as opposed to 1.96±0.13 kPa in healthy lung tissue (Booth et al., 2012). A stiffer matrix also promotes IPF-like phenotypes, such as enhanced differentiation, proliferation and resistance to apoptosis in non-IPF fibroblasts (Liu et al., 2010; Marinkovic et al., 2012; Paszek et al., 2005; Wipff et al., 2007). Despite the availability of two recently approved drugs for slowing the progress of IPF [nintedanib (Mazzei et al., 2015; Richeldi et al., 2014a,b) and pirfenidone (King et al., 2014; Valeyre et al., 2014)], their associated incidence of side effects and modest clinical benefit warrants further investigation into possible new therapeutic directions for IPF (Moodley et al., 2015). Selective inhibition of LOXL2 activity was recently investigated as a therapeutic approach for IPF [Barry-Hamilton et al., 2010; ClinicalTrials.gov NCT01769196 (Raghu et al., 2017)]. Although this trial was terminated early because of lack of efficacy, there is still potential for the development of effective LO inhibitors for targeting fibrosis. To enhance understanding of the mechanisms by which LOX and LOXL enzyme inhibition might be beneficial in IPF, this study aimed to investigate fibrillar collagen structural remodelling in IPF lung tissues and to determine the expression profile of LOs in IPF. It also investigated the effectiveness of pan-LO and selective LOXL2 inhibition on fibrillar collagen structural remodelling and tissue stiffness in the context of IPF.

## RESULTS

### Increased fibrillar collagen maturity/organization in IPF lung tissues

**Fig. 1.**
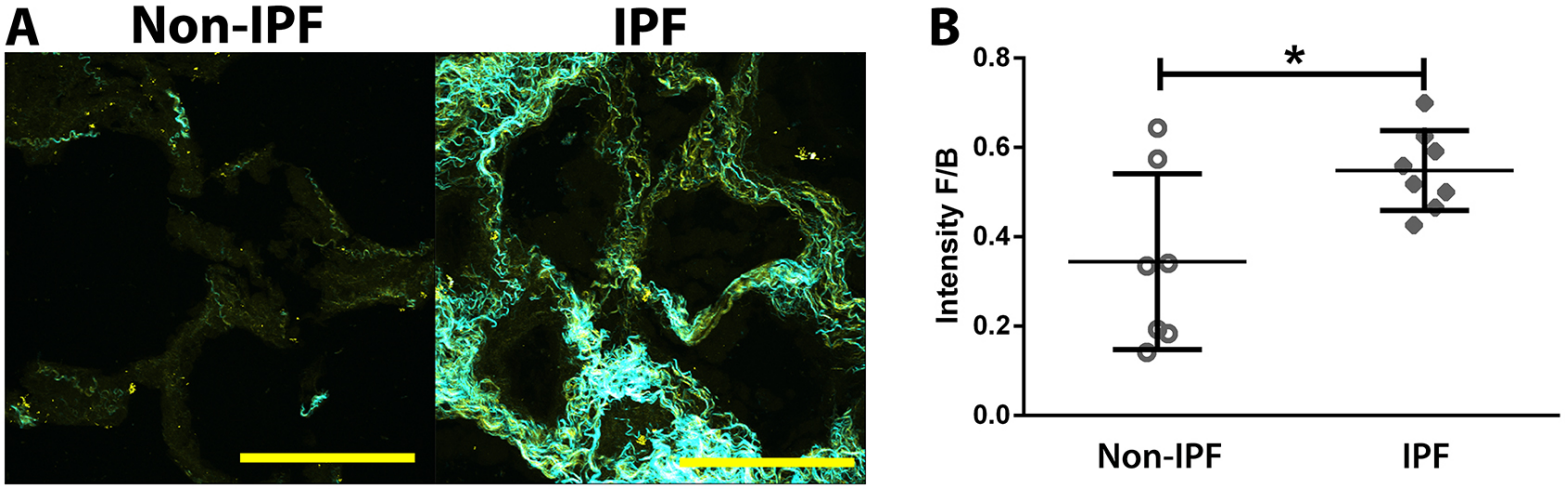
**Increased level of collagen organization/maturity in IPF lung parenchyma.** (A) Representative SHG images of lung parenchyma from non-IPF (*n*=7; open circles) and IPF (*n*=8; filled diamonds) subjects (average of three or four samples per subject; yellow indicates backward immature/disorganized collagen; cyan indicates mature/organized collagen; scale bar: 100 µm). Brightness and contrast have been enhanced for display purposes only and were applied equally for all images. (B) Image analysis quantification of SHG intensity forward/backward signal ratio (intensity F/B; each point represents the mean of three or four lung parenchymal samples per subject); a higher ratio indicates higher proportion of organized/mature fibrillar collagen. Data were analysed with Student's two-tailed parametric unpaired *t*-test. Data are presented as means±s.d. Data were obtained from one experimental replicate per subject. *P<0.05.

To investigate the collagen structural remodelling in IPF, SHG microscopy was performed on lung tissue sections from non-IPF and IPF subjects. The SHG forward (F) signal is predominantly from mature/organized fibrillar collagen, whereas the backward (B) signal is from immature/disorganized fibrillar collagen. Thus, increased F/B area corresponds to increased amounts of mature/immature collagen, whereas increased F/B intensity ratios correspond to an increased degree of collagen maturity/thickness and vice versa (Tjin et al., 2014). Quantification of SHG images showed that IPF lung parenchymal tissue had greater levels of fibrillar collagen maturity/organization compared with the non-IPF tissues (Fig. 1A,B). By contrast, the collagen structural organization was not different between IPF and non-IPF ILD tissues (Fig. S1).

### Lysyl oxidase genes were differentially expressed in IPF and healthy control lung tissues

Fibrillar collagen maturity is catalysed by the LO family of enzymes. To investigate the levels of LO enzymes in lung tissues, lung tissue gene expression profiles of IPF and healthy controls were analysed from two publically available data sets (Table 1). *LOXL1* gene expression was increased in IPF lung tissue compared with healthy controls in two independent studies [false discovery rate (FDR) adjusted *P*-value <0.25], whereas the *LOXL2* gene expression was increased in IPF lung tissue in a single data set. No differences were found in *LOX* gene expression levels.

**Table 1.**
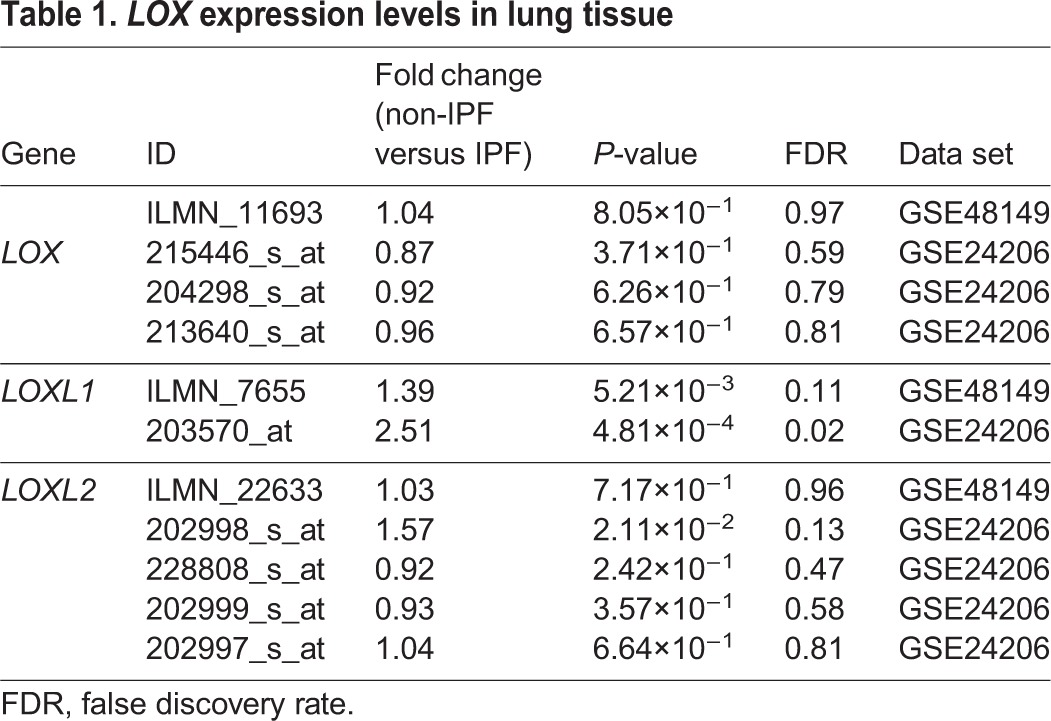
***LOX* expression levels in lung tissue**

### Lysyl oxidase protein levels were differentially expressed between IPF and non-diseased lung tissues

**Fig. 2.**
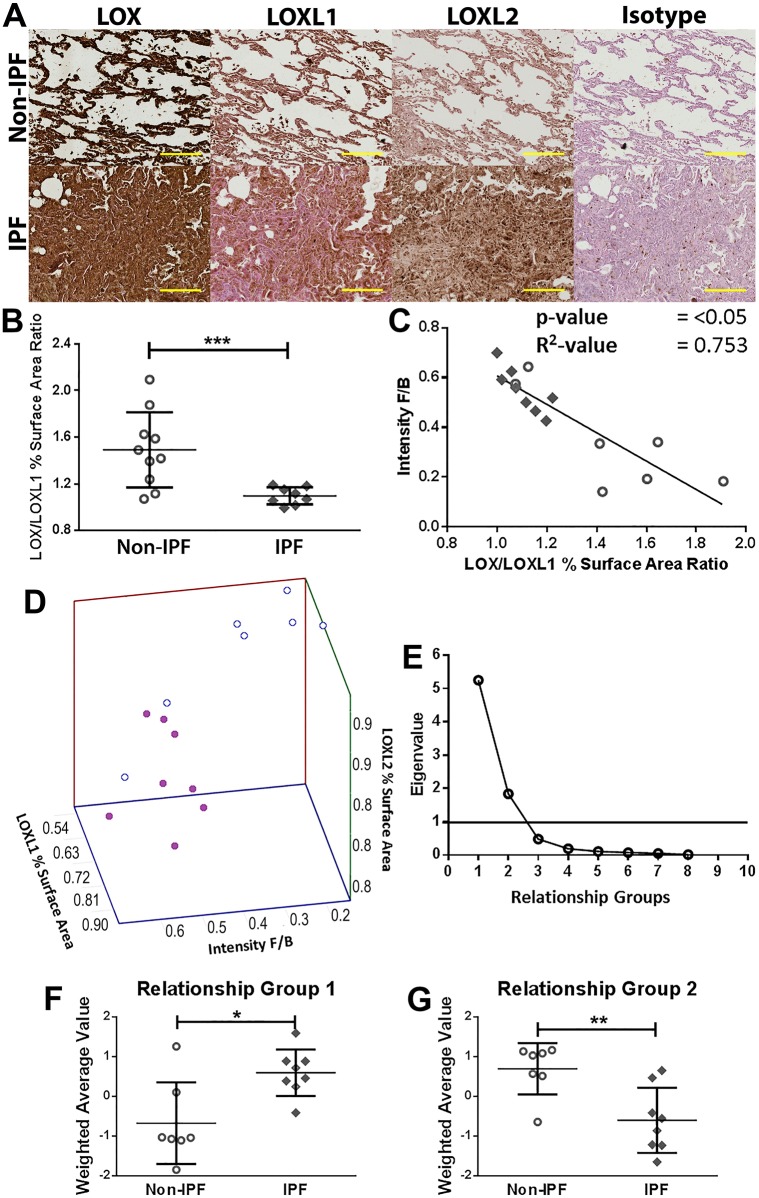
**Collagen organization and lysyl oxidase family of enzymes are differentially expressed and** **interrelate** **differently in IPF.** (A) Representative IHC images of lysyl oxidase enzymes in the lungs. Images are representative of lung tissues from non-IPF (*n*=10) and IPF (*n*=8) subjects (brown, protein of interest; blue, nucleus; pink, cytoplasm; scale bar: 200 µm). (B) The ratio of LOX and LOXL1 percentage surface area obtained by image analysis of IHC-stained lung tissues of non-IPF (*n*=10; open circles) compared with IPF (*n*=8; filled diamonds) subjects; data were analysed with Student's two-tailed parametric unpaired *t*-test. (C) Correlation of the ratio of LOX and LOXL1 with F/B ratio obtained through image analysis of SHG microscopy images of collagen structure in non-IPF (*n*=7) and IPF (*n*=8) subjects (*P*<0.0001; *R*^2^=0.7527; average of three or four samples per subject). (D) 3D scatter plot of collagen organization (intensity F/B, i.e. SHG intensity forward/backward signal ratio), LOXL1 and LOXL2 expression (open circles, non-IPF, *n*=7; filled circles, IPF, *n*=8). (E) Scree plot showing the number of relationship groups needed to explain the associations within measured variables in non-IPF and IPF tissues (eigenvalue threshold≥1). (F,G) Weighted average values of the variables contributing to each relationship group; comparing non-IPF with IPF subjects (average of three or four samples per subject); data were analysed with Student's two-tailed parametric unpaired *t*-test. Data are presented as means±s.d. Data were obtained from one experimental replicate per subject. *P<0.05, **P<0.01, ***P<0.005.

To confirm the findings from the gene expression study, tissue sections from the same samples used for the examination of collagen structural organization were examined for LOX, LOXL1 and LOXL2 protein levels. Quantification of the captured images of the stained lung tissue sections showed that LOX percentage surface area and density were decreased in IPF compared with non-IPF, whereas the percentage surface area and density of LOXL1 was increased in IPF compared with non-IPF tissues (Table 2; representative images in Fig. 2A). In agreement with prior studies (Aumiller et al., 2017; Barry-Hamilton et al., 2010), LOXL2 density was also increased in IPF compared with non-IPF tissues. By contrast, only LOXL1, but not LOX or LOXL2, density increased in IPF compared with non-IPF ILD (Table S1). *In vitro*, primary human lung parenchymal fibroblasts from IPF patients produced significantly greater levels of LOXL2 but not LOX or LOXL1 in the cell lysate compared with non-IPF controls (Fig. S2A,B).

**Table 2.**
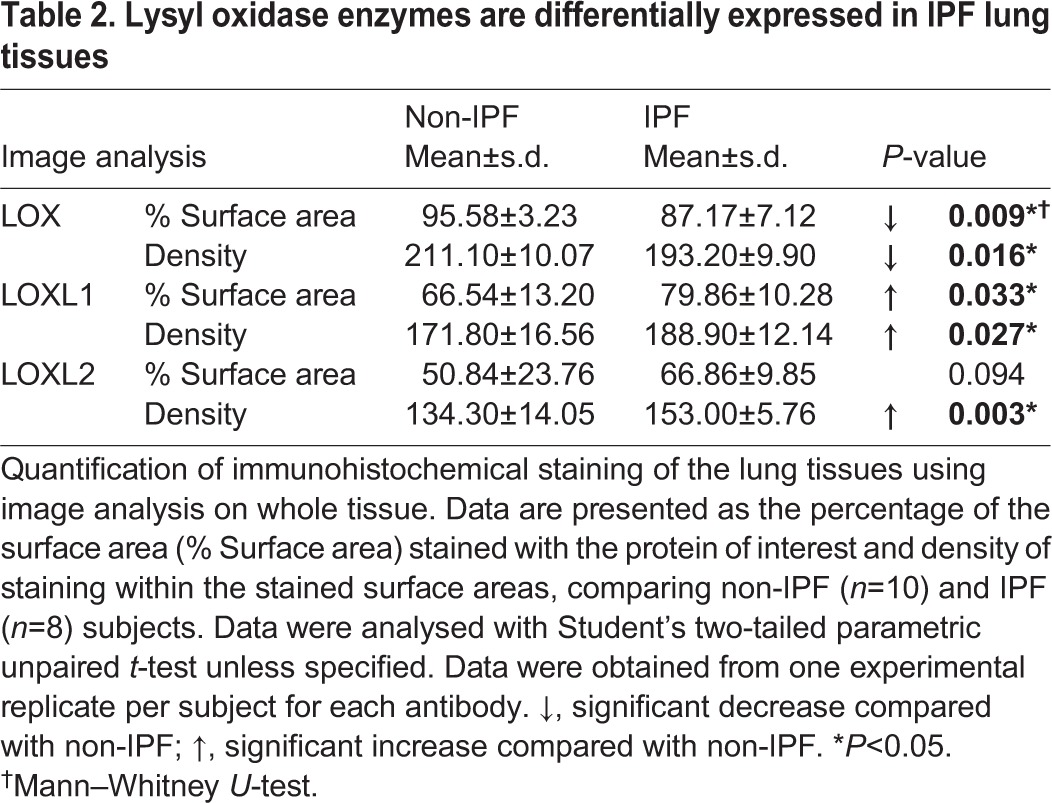
**Lysyl oxidase enzymes are differentially expressed in IPF lung tissues**

### Expression of LOs was correlated with collagen remodelling in lung tissues

To begin to understand the relationship between LOs and collagen structural arrangement in lung tissues, we used correlation analysis. The data from both non-IPF and IPF subjects were combined in the correlation studies to examine the whole spectrum of collagen remodelling in both cohorts. There was a positive correlation between both LOXL1 (Table 3; Fig. S3) and LOXL2 (Table 3) with collagen remodelling (SHG F/B ratios), whereas there was a negative correlation between LOX and SHG forward to backward signal (F/B) ratios (Table 3). LOXL1 positively correlated with LOXL2, whereas LOX negatively correlated with both LOXL1 and LOXL2 (Table 3).

**Table 3.**
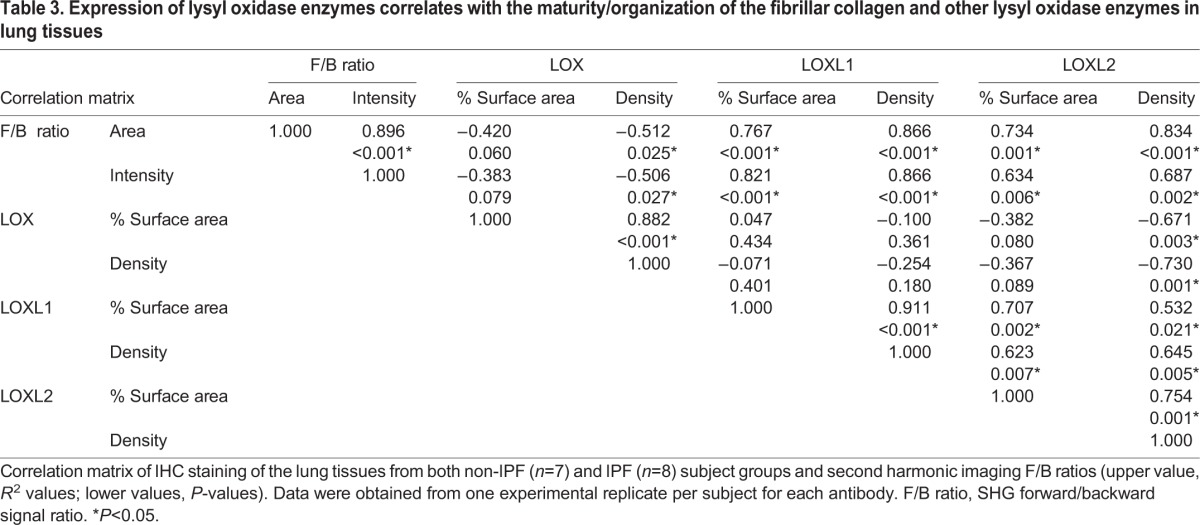
**Expression of lysyl oxidase enzymes correlates with the maturity/organization of the fibrillar collagen and other lysyl oxidase enzymes in lung tissues**

### Lysyl oxidase family members were differentially expressed in IPF

The ratio of LOX/LOXL1 (Fig. 2B) and LOX/LOXL2 (Fig. S4) densities and LOX/LOXL1 percentage surface areas were significantly decreased in IPF lung tissue samples compared with non-IPF subjects (Fig. 2B; Fig. S4). The ratio of LOX/LOXL1 percentage surface area inversely correlated with collagen maturity/organization (F/B; Fig. 2C). There was no significant difference in the ratios of LO family enzymes in the tissues of IPF and non-IPF ILD subjects (Fig. S5; *P*=NS).

### Collagen organization and LOX enzymes interrelate differently in IPF

Given that LOX, LOXL1 and LOXL2 were differentially expressed in IPF compared with non-IPF (Fig. 2B,C) and the expression of these enzymes also correlated strongly with collagen remodelling (Table 3), it was important to understand the interrelationship between these parameters in IPF. Visualization of the three-dimensional (3D) association among these variables showed spatial separations between the variables in IPF compared with non-IPF (Fig. 2D). We next performed factor analysis to investigate the number of relationship groups (factors) accounting for the structure of these associations. A minimum of two groups were required to explain the associations within the data set (Fig. 2E), and Table S2 shows the strength of the contribution of the variables [LOX, LOXL1, LOXL2, immunohistochemistry (IHC) data and SHG data] to the two relationship groups. The strengths of contributions of the variables were used to determine the weighted average values for each relationship group (Fig. 2F,G). The values for relationship Groups 1 and 2 were significantly different between non-IPF and IPF, indicating a difference in the relationships between the variables (LOX, LOXL1, LOXL2 IHC data and SHG data) in disease.

### Inhibition of LO activity reduced TGF-β-induced collagen remodelling in collagen I hydrogels

**Fig. 3.**
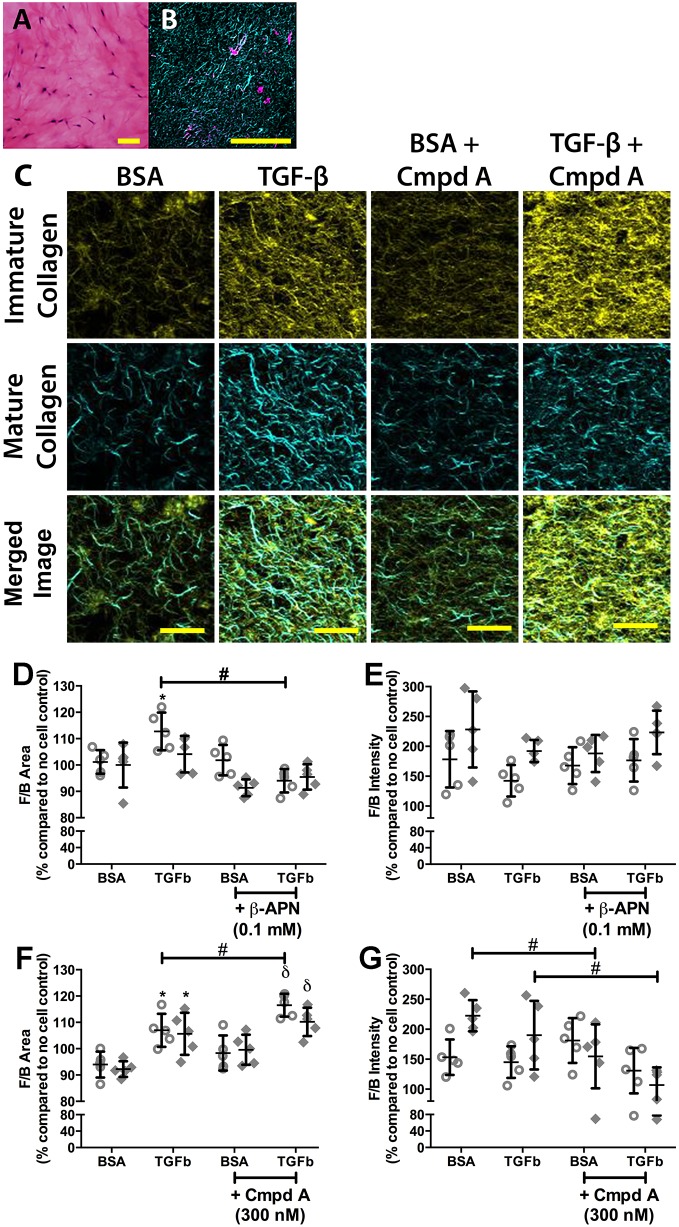
**Inhibition of lysyl oxidase activity reduces TGF-β-induced collagen remodelling.** Collagen I hydrogels seeded with primary human lung parenchymal fibroblasts (250,000 cells ml^−1^) were stimulated with TGF-β (10 ng ml^−1^) to induce a fibrotic phenotype in the presence or absence of the pan-lysyl oxidase inhibitor, β-APN (100 µM), or the LOXL2-specific inhibitor, Compound A (Cmpd A; 300 nM). Samples were treated for 7 days. At the end of the treatment period, the samples were formalin fixed and paraffin embedded. (A) Haematoxylin and Eosin stain of fibroblasts in collagen gel (blue, nucleus; pink, cytoplasm and general tissue stain; scale bar: 100 µm) at day 7. (B) SHG image of collagen gel with fibroblasts at day 7 (cyan, forward mature/organized collagen; magenta, cell autofluorescence; scale bar=100 µm). (C) Representative SHG images of collagen gels with primary human lung parenchymal fibroblasts treated with or without TGF-β (10 ng ml^−1^) in the presence or absence of Compound A (300 nM) (yellow, backward immature/disorganized collagen; cyan, forward mature/organized collagen; scale bar: 20 µm). (D-G) Quantification of SHG microscopy on collagen I hydrogels embedded with fibroblasts from non-IPF (*n*=5; open circles) and IPF (*n*=5; filled diamonds) subjects treated with pan-lysyl oxidase inhibitor (β-APN; D,E) or LOXL2-specific inhibitor (Compound A; F,G). Data are presented as a percentage of the respective no-cell control (means±s.d.). (D,F) SHG forward/backward signal surface area ratio (F/B area); higher ratio indicates higher amount of mature compared with immature collagens. (E,G) SHG forward/backward signal intensity ratio (F/B intensity); higher ratio indicates greater maturity/thickness level of fibrillar collagen fibres. Statistical analysis was by two-way ANOVA with matching and multiple comparisons with Tukey's correction. Data are presented as means±s.d. Data were obtained from one experimental replicate per subject. Brightness and contrast have been enhanced for display purposes only and were applied equally for all images. *Significant difference compared with BSA control; ^δ^significant difference compared with BSA+Compound A control; ^φ^significant difference compared with TGF-β alone; and ^#^significant difference between groups.

To confirm the role of LO enzymes in fibrillar collagen remodelling, 3D *in vitro* cell culture experiments using inhibitors of LO activity were performed. β-APN is a non-selective inhibitor of LO activity, whereas Compound A is a specific inhibitor of LOXL2 activity (Schilter et al., 2014). Pan-inhibition of LO activity, but not inhibition of LOXL2-specific activity, reduced TGF-β-induced collagen remodelling in collagen hydrogel fibroblast cultures (Fig. 3). TGF-β increased the deposition of mature fibrillar collagen by non-IPF fibroblasts but not IPF fibroblasts, which was alleviated by the pan-LO inhibitor β-APN (Fig. 3D), but TGF-β did not affect collagen fibre maturity/thickness (Fig. 3E). By contrast, Compound A did not alleviate the increased deposition of mature collagen compared with immature collagen, from both fibroblast groups, induced by TGF-β, but on the contrary, increased it (Fig. 3F). Compound A did reduce the thickness of the fibres generated by the IPF fibroblasts in the presence and absence of TGF-β stimulation (Fig. 3G).

### Inhibition of LO activity reduced TGF-β-induced collagen remodelling in reseeded decellularized matrices

**Fig. 4.**
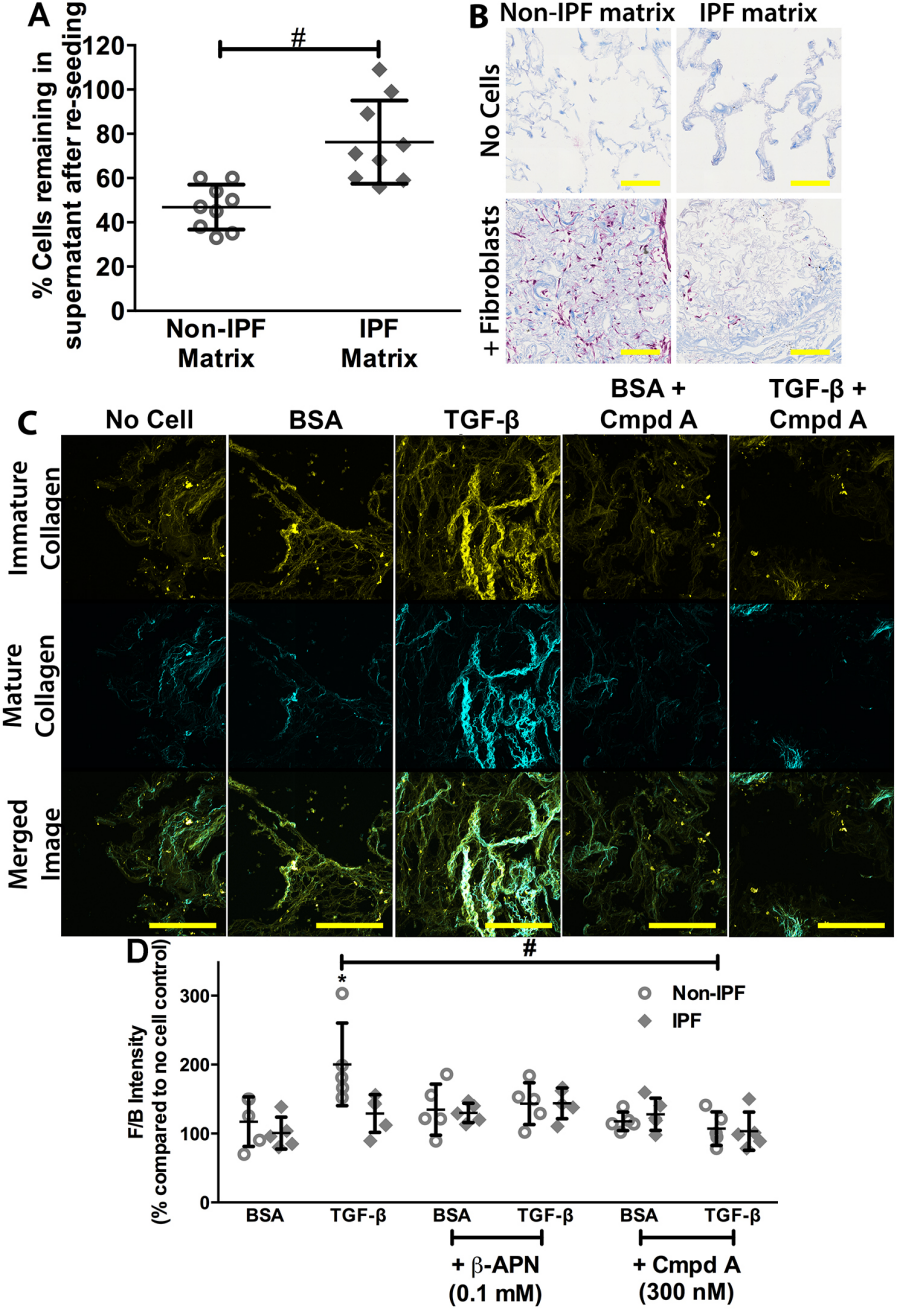
**Inhibition of lysyl oxidase activity reduces TGF-β-induced collagen remodelling.** Decellularized non-IPF matrices reseeded with primary human lung parenchymal fibroblasts (1×10^6^ cells per matrix) were stimulated with TGF-β (10 ng ml^−1^) to induce a fibrotic phenotype in the presence or absence of the pan-lysyl oxidase inhibitor, β-APN (100 µM), or the LOXL2-specific inhibitor, Compound A (Cmpd A; 300 nM). Cells were seeded in the matrix for 24 h before being transferred into separate tissue culture wells with treatment media for 7 days. Samples were collected at the end of the treatment period and formalin fixed and paraffin embedded. (A) Manual cell counts of cells remaining in supernatant after reseeding in matrices [*n*=9; combined data from 4 non-IPF and 5 IPF fibroblasts (500,000 cells ml^−1^; 2 ml per matrix) into matrices from one non-IPF and one IPF subject]. (B) Masson's Trichrome stain of fibroblasts reseeded into decellularized matrices for 7 days (blue, collagen; pink, cell cytoplasm; scale bar: 100 µm). (C) Representative SHG images of collagen gels with fibroblasts treated with or without TGF-β (10 ng ml^−1^) in the presence of or absence of Compound A (300 nM) (yellow, backward immature/disorganized collagen; cyan, forward mature/organized collagen; scale bar: 100 µm). (D) Quantification of SHG microscopy on decellularized non-IPF matrices reseeded for 7 days with fibroblasts from control (*n*=5; open circles) and IPF (*n*=5; filled diamonds) subjects treated with β-APN (100 µM) or Compound A (300 nM) in the presence or absence of TGF-β (10 ng ml^−1^). Data are presented as a percentage of the respective no-cell control (means±s.d.); SHG forward/backward signal intensity ratio (F/B intensity); higher ratio indicates greater maturity/thickness level of fibrillar collagen fibres. Statistical analysis was by two-way ANOVA with matching and multiple comparisons with Tukey's correction. Data are presented as means±s.d. Data were obtained from one experimental replicate per subject. Brightness and contrast have been enhanced for display purposes only and were applied equally for all images. *Significant difference compared with BSA control; and ^#^significant difference between groups.

To model the *in vivo* cell ECM interactions more closely, we used decellularized human IPF and non-IPF lung scaffolds. There were significantly more primary human lung fibroblasts remaining in the supernatant after reseeding of the IPF matrices compared with the non-IPF matrices (Fig. 4A), potentially indicating higher cell attachment to the non-IPF matrix. This was supported by histological staining of the reseeded matrices, which showed that fewer cells were detectable in the IPF matrix post-reseeding, compared with the non-IPF matrix (Fig. 4B). Given the sparsity of cellular attachment in the IPF matrices, these matrices were not examined further.

The non-IPF cells increased the maturity/thickness of the collagen fibres in the non-IPF matrix in the presence of TGF-β (Fig. 4C,D) but did not increase the amount of mature collagen compared with immature collagen (Fig. S6). The pan-inhibitor of LOs, β-APN, returned TGF-β-induced collagen remodelling to baseline. In addition, the LOXL2-specific inhibitor blocked TGF-β-induced remodelling of fibrillar collagen; remodelling levels in the presence of either inhibitor without TGF-β were not different compared with control (Fig. 4D). Primary IPF lung fibroblasts seeded into decellularized normal matrices did not remodel the fibrillar collagen in the presence of TGF-β (Fig. 4D).

### Inhibition of LO activity reduced TGF-β-induced stiffening in reseeded decellularized matrices

**Fig. 5.**
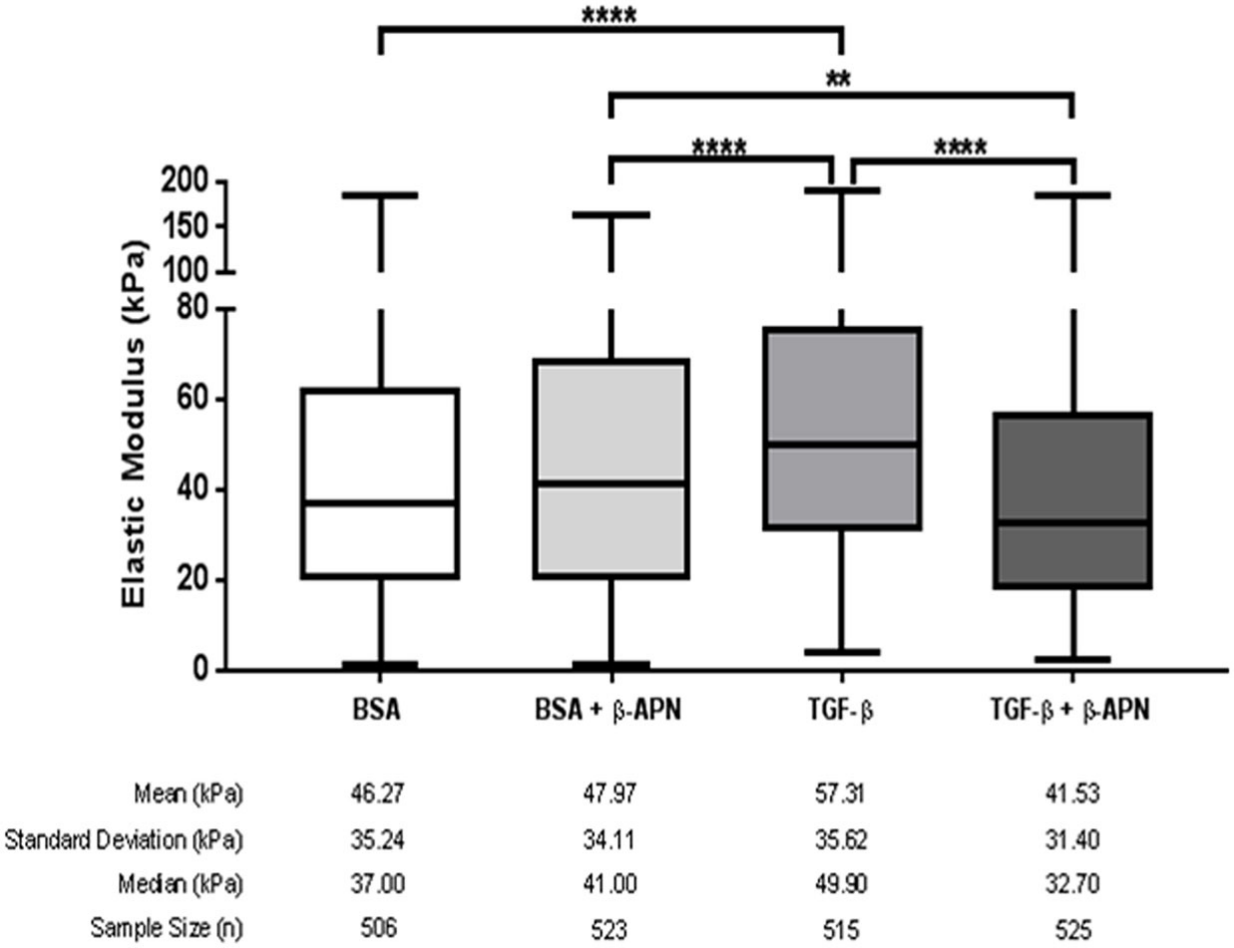
**Inhibition of lysyl oxidase activity reduces TGF-β-induced stiffening of reseeded decellularized matrices.** Decellularized non-IPF matrices reseeded with primary human lung parenchymal fibroblasts (10^6^ cells per matrix) were stimulated with TGF-β (10 ng ml^−1^) in the presence or absence of the pan-lysyl oxidase inhibitor, β-APN (100 µM). Cells were seeded in the matrix for 24 h before being transferred into separate tissue culture wells with treatment media for 7 days. Samples were collected at the end of the treatment period and fresh frozen in OCT compound (10 µm slices). Box and whiskers plot of the stiffness (elastic modulus). Stiffness was measured with AFM, and >100 measurements spread over three areas were taken per sample. Each *n* is a single AFM measurement. Data were obtained from one experimental replicate per subject per treatment. Statistical analysis was by the Kruskal–Wallis test with Dunn's correction. **P<0.01, ****P<0.0001.

To confirm the functional significance of TGF-β-induced collagen remodelling in the decellularized matrices reseeded with non-IPF cells, tissue stiffness (elastic modulus) was measured by atomic force microscopy (AFM; micro-indentation). TGF-β induced a significant increase in tissue stiffness that was inhibited by the pan-inhibitor of LOs, β-APN (Fig. 5). Individual stiffness measurements for each patient segregated by treatment can be found in Fig. S7, and representative force curves used to measure stiffness can be found in Fig. S8C.

## DISCUSSION

Although increased collagen deposition is well appreciated in fibrotic lung tissues, this study identifies, for the first time, alterations in the fibrillar organization of the collagen fibres in IPF lung tissue. In addition, this study reports that *LOXL1* and *LOXL2* gene and protein levels are increased in the lungs of IPF compared with non-IPF subjects, with a positive correlation between increased protein expression and increased fibrillar collagen organization.

These findings show the complex interrelationship between LOs and collagen structure and provide further support for focusing on LO inhibition as a potential therapeutic approach in IPF. Indeed, LOXL2 inhibition prevented and even reversed bleomycin-induced pulmonary fibrosis in mice (Barry-Hamilton et al., 2010). In addition, LOX and LOXL2 were also found to be elevated in liver fibrosis (Barry-Hamilton et al., 2010; Vadasz et al., 2005) and were associated with increased deposition of collagen around the hepatocytes (Vadasz et al., 2005). Although the recent clinical trial targeting LOXL2 inhibition (ClinicalTrials.gov NCT01769196) was halted early because of a lack of efficacy (Raghu et al., 2017), the present study suggests that targeting LOXL1 in IPF might be as important as targeting LOXL2.

In the present study, IPF alveoli had higher collagen maturity/thickness (increased F/B SHG) compared with non-diseased subjects. It can be postulated that the increased collagen fibre maturity/thickness contributes to the increased stiffness characteristic of IPF lungs (Booth et al., 2012). This is supported by the increased stiffnesses measured in this study in the decellularized lung scaffolds reseeded with fibroblasts exposed to TGF-β. In IPF there is augmented TGF-β (Broekelmann et al., 1991), which induces IPF fibroblasts to produce enhanced ECM proteins (Westergren-Thorsson et al., 2004), thereby creating a positive feedback loop. In fact, collagen cross-linking might account for organ stiffening in a variety of fibrotic diseases. The results of the present study are also concordant with those of Kottmann et al. (2015), who demonstrated an altered F/B SHG signal in IPF compared with non-diseased lungs. Interestingly, the study by Kottmann et al. (2015) showed a decrease in F/B SHG between healthy and IPF lungs; this difference might be attributable to different sampling and analysis protocols in comparison to the present study. For example, in the study by Kottmann et al. (2015), the majority of the F SHG signal (numerator in the F/B SHG signal ratio) from healthy subjects originated from muscular arteries, whereas the present study excluded such regions from analysis. That said, neither study identified differences in collagen remodelling between IPF and other fibrotic interstitial lung diseases.

A potential role of other LO family members, not only LOXL2, in collagen remodelling in IPF was also highlighted in the present study. A significant increase in *LOXL1* gene and protein expression in IPF lungs, compared with non-IPF tissues, was observed, with a positive correlation of protein levels with collagen organization. By contrast, *LOX* gene expression was unchanged, whereas the LOX protein level was decreased in IPF lung, highlighting differential LO family expression in IPF lung tissue, which is similar to that reported during tumour progression and metastasis (Erler et al., 2006; Payne et al., 2005). In concordance with our findings, a recent study by Aumiller et al. (2017) also reported increased LOXL1 and LOXL2 protein levels in IPF lungs compared with healthy lungs. By contrast, however, they also reported an increased LOX protein level in IPF (Aumiller et al., 2017). These differential results might be attributable to the different analysis approaches. Aumiller et al. (2017) scored the intensity of LO staining in discrete structures of the lung, whereas in our study we used digitized image analysis to measure the staining intensity of the whole lung tissue section whilst controlling for the increased tissue mass in IPF lungs.

Of the LO family enzymes, only the LOXL1 protein level was increased in IPF compared with non-IPF ILD lungs, indicating that changes in LOXL1 might be specific to IPF rather than to the process of fibrosis itself. To our knowledge, this study is the first to show a difference in the interrelationships of the organization of collagen, LOX, LOXL1 and LOXL2 enzymes between the lungs of IPF and non-IPF subjects. This is particularly important, because a Phase II clinical trial using simtuzumab, a LOXL2-specific inhibitor [ClinicalTrials.gov NCT01769196 (Raghu et al., 2017)] was recently terminated early owing to a lack of efficacy. The data presented in the present study support the continued development of new therapeutic strategies targeting LOs for IPF, especially focusing on exploration of the potential of LOXL1-specific inhibitors or combination approaches.

To understand the role of LOs in the remodelling of collagen fibres in IPF, two 3D *in vitro* culture models were used to mimic the complexity of the matrix *in vivo*. Interestingly, the decellularized IPF matrices supported lower primary fibroblast infiltration and viability compared with non-IPF matrices. Although prior works (Booth et al., 2012; Parker et al., 2014) are in line with the present data that decellularized IPF matrices influence cellular phenotype, previous evaluations had not examined differences in cell attachment between non-IPF and IPF matrices. Thus, these observations highlight another difference between IPF and non-IPF lung matrices that should be investigated as a contributor to the pathogenesis of fibrosis. These findings further highlight the challenge ahead in finding solutions for repairing the disrupted ECM in IPF.

In collagen gels, TGF-β induced fibroblast production of mature/thick collagen fibres, but did not affect the overall maturity/thickness of the fibres. By contrast, TGF-β did not significantly alter the amount of mature collagen fibres in decellularized human lung matrices, whereas overall maturity/thickness was increased. This apparent paradox might simply be attributable to the model compositions, in that collagen hydrogels lack the complex ECM components found in decellularized matrices, components that might influence cellular responsiveness. Alternatively, the artificiality of the collagen hydrogel, where collagen fibres are not in a native structural configuration, leads cells to respond by depositing and remodelling collagen fibres to attain a native environment. By contrast, decellularized matrices are composed of collagen fibres and ECM already in a native configuration, thereby driving cellular driven ECM remodelling rather than production. This latter conclusion is supported by the presented data (Figs 4 and 5) showing collagen gel F/B intensity >150% of the no cell control even in unstimulated conditions, whereas decellularized matrices were recorded at ∼100% in the same conditions. The differences in the presence of artificial matrix (collagen hydrogel) and native matrix (decellularized matrix) support previous findings that the ECM contributes a very important regulatory control on cellular behaviour (Booth et al., 2012; Parker et al., 2014).

Pan-inhibition of LO activity blocked TGF-β-induced collagen remodelling in both collagen hydrogels and decellularized matrix systems. By contrast, LOXL2-specific inhibition reduced collagen fibril maturity/thickness only in IPF fibroblasts and in decellularized matrices. As this effect was not seen with the pan-inhibitor of LOs, it is likely that this effect was independent of LOXL2. However, prior data demonstrate that fibroblasts grown in two-dimensional (2D) culture express a 50 kDa proteolytically processed form of LOXL2 (Hollosi et al., 2009), rather than the intact 95 kDa LOXL2 enzyme that is inhibited by Compound A. Although the functional activity of the LOXL2 50 kDa isoform is unknown, it seems unlikely to account for the present observations, because Compound A was able to block other TGF-β-induced effects in 2D and 3D culture. Additionally, pan-inhibition of LO activity blocked TGF-β-induced tissue stiffening in the decellularized matrix system (Fig. 5). Both collagen fibril maturity/thickness and tissue stiffness were increased by TGF-β treatment, with both being interrupted in the presence of β-APN. These data indicate that LO enzymes regulate the TGF-β-induced increase in collagen fibril maturity/thickness that results in increased tissue stiffness. The pro-fibrotic features of stiff matrices contribute to the TGF-β-induced fibrosis with potential feedback mechanisms through the promotion of latent TGF-β activation (Wipff et al., 2007), thus contributing to the fibrotic process in IPF.

It is worth noting that the stiffness measured in the reseeded decellularized matrices (mean 40-60 kPa) was higher than the values measured in native human lung parenchymal tissue (1.34±0.36 kPa; Liu et al., 2010) and IPF tissue (16.52±2.25 kPa; Booth et al., 2012). It has been reported in the literature that the decellularization method affects the stiffness of acellular tissue (Melo et al., 2014) and, more generally, that decellularized tissue is stiffer than normal lung tissue (Giménez et al., 2017), although the origins of these differences in decellularized versus normal tissue stiffness remain poorly understood. The reseeded decellularized matrices did not form alveolar structures in the time span of this study and were densely packed (Fig. 4B), possibly as a result of lack of inflation of the tissues, which is not possible within the present experimental set-up. This tissue structure might more closely mimic scar tissue or remodelled IPF lung tissue (characterized by progressive scarring of the lungs) rather than non-diseased lung tissues. In such a case, this would strengthen the significance of reseeded decellularized matrices in the study of IPF tissue remodelling. It should also be noted that β-APN might have non-cell-induced effects on the stiffness, as the stiffness appears lower in β-APN-treated non-reseeded matrices than those without β-APN treatment (∼40 versus ∼60 kPa, respectively; Fig. S7), but the present experiment was not designed to address this hypothesis.

Overall, this study highlights a potential mechanism for preventing ongoing fibrotic processes, with LO inhibition preventing lung fibrogenesis in IPF by blocking fibrillar collagen organization and subsequent tissue stiffening. This, in turn, might leave deposited collagen more susceptible to proteolytic degradation and clearance, although further research is needed to investigate this possibility. The present findings also highlight the role of multiple members of the LO family in regulating collagen cross-linking and the disrupted balance of LOs in the IPF lung. These findings indicate that selective inhibition of LO enzymes, such as LOXL1, either alone or in combination with LOXL2 inhibition, might be a potential therapeutic strategy for targeting fibrosis in IPF.

## MATERIALS AND METHODS

### Human tissues

Human lungs were procured with written and informed consent from the patients or next of kin from non-transplantable donors and IPF patients undergoing lung transplantation as previously described (Booth et al., 2012). Approval for all experiments with human lung tissue was provided by the Ethics Review Committees of the South West Sydney Area Health Service, St Vincent's Hospital Sydney, Royal Prince Alfred Hospital (RPAH), and the University of Sydney Human Research Ethics Committee. As tissues were de-identified, the University of Michigan Institutional Review Board deemed this work exempt from oversight. Primary human lung fibroblast samples used as non-diseased controls (non-IPF) were from the macroscopically normal regions of lungs from patients with non-small cell carcinoma (NSCCa). Human lung tissue samples were classified by pathologist diagnosis. Patient demographics are described in Table S3.

### Tissue and cell isolation

Tissue isolation was performed on fresh explanted lung tissues. Human lung parenchymal tissues were isolated and cut into pieces no bigger than 1 cm^3^. These pieces were fixed in 4% formaldehyde (Fronine; Thermo Fisher Scientific, Waltham, MA, USA). After formaldehyde fixation, isolated lung tissues were embedded in paraffin.

Primary human lung fibroblasts were isolated as previously described (Krimmer et al., 2012; White et al., 2003) and were grown in growth media [Dulbecco's modified Eagle's medium (DMEM) low glucose (Gibco, Thermo Fisher Scientific) with 0.025 M HEPES (Gibco), 0.375% sodium hydrogen carbonate (Ajax Finechem, Thermo Fisher Scientific), 10% (v/v) FBS (JRH Biosciences, Brooklyn, Victoria, Australia) and 1% (v/v) antibiotics (Invivogen, San Diego, CA, USA), pH 7.1-7.2]. All fibroblast cell lines were tested for mycoplasma contamination after cell isolation and culture. For experiments, mycoplasma-free fibroblasts were used between the third and ninth passages.

### *In vitro* cell culture

Primary human lung fibroblasts were grown in growth media [DMEM low glucose (Gibco) with 0.025 M HEPES (Gibco) and 0.375% sodium hydrogen carbonate (Ajax Finechem, Thermo Fisher Scientific)] incubated in a humidified CO_2_ incubator (5% CO_2_ in air) at 37°C. Growth media were aspirated from cells every 4 days and replaced with fresh growth media until confluence.

### Protein extraction from primary human lung fibroblasts

Whole-cell lysates were prepared using protein extraction buffer [20 mM Tris (Amresco, Dallas, TX, USA) pH 7.4 with 150 mM sodium chloride (PanReac Applichem, Darmstadt, Germany), 1 mM EGTA (Sigma, St Louis, MO, USA), 1 mM EDTA (Amresco), 1 mM sodium fluoride (NaF; Sigma), 20 mM sodium pyrophosphate (Sigma), 2 mM sodium orthovanadate (Na_3_VO_4_; Sigma), 1% (v/v) Triton X-100 (Sigma), 10% (v/v) glycerol (Sigma), 0.1% (w/v) SDS (Amresco), 0.5% (w/v) sodium deoxycholate (Sigma), 1 mM PMSF (Life Technologies, Solon, OH, USA), 1% (v/v) Protease Inhibitor Cocktail Set III (Calbiochem, San Diego, CA, USA)] on ice, scraped into a tube, and centrifuged at 150×***g*** for 5 min. The supernatants were collected and stored at −80°C for further experiments. Total sample protein was measured using a standard BCA assay (Sigma).

### SHG microscopy

SHG imaging and analysis were performed on paraffin-embedded, formalin-fixed lung tissue sections as previously published (Tjin et al., 2014).

Thirty-micrometre-thick samples were used for SHG analysis in human tissue samples (cohort 1, non-IPF=7 versus IPF=8; and cohort 2, non-IPF ILD=8 versus IPF=26) and reseeded decellularized human lung tissue samples. Five-micrometre-thick tissue samples were used for the SHG in collagen gel samples, as the softness of the sample prevented the use of thicker samples because of the unavoidable compression of these samples during processing. Confocal images were taken at ×63 magnification at 16-bit resolution for both forward and backward propagated SHG signals, with identical detector configurations for all images taken. Laser power was measured at the objective before the start of each experiment performed on different days, and laser power settings were adjusted so that all experiments use the same laser power (25 mW).

Owing to the heterogeneous nature of the lung tissues, a 10×10 grid was applied on the human tissue samples using the tile function of the Leica LAS AF software (Leica, Wetzlar, Germany). Three regions for imaging were then selected using a random number generator. A 5×5 grid was used on decellularized tissue samples because of the smaller size of the samples compared with the size of human lung tissue samples. Collagen gel samples were more homogeneous, and three regions were randomly selected for SHG.

In the figures, adjustments of brightness and contrast as indicated in the figure legends were applied uniformly for every set of images and were used for display purposes only.

### SHG image analysis

Image analysis was performed using Fiji (Schindelin et al., 2012) with images imported from the LAS AF software (Leica). The stack was deliberately oversampled by 20% in each direction to ensure that the entirety of the tissue section was imaged. The confocal 3D image was analysed using the histogram to determine the mean (µ) and s.d. (σ) of the signal for the image. A threshold of µ+σ was then applied to the image to exclude background emissions as previously published (Abraham et al., 2012).

The pixel area (the number of pixels with intensity above threshold) and pixel density (average signal intensity per pixel with intensity above threshold) was measured for every slice in the image using batch processing. The total signal intensity (total intensity for all pixels with intensity above threshold) was determined as the product of the pixel area and density. The average values for these measurements were calculated for every image in the stack. The above process was repeated for both forward and backward propagated data to generate area, intensity and density data for both forward and backward propagated signals. The ratio of forward to backward signal was then calculated for area, intensity and density measurements.

### Lysyl oxidase gene expression in lung tissues

Two publically available microarray data sets from lung tissue samples of IPF patients and healthy controls were analysed (GSE48149, non-IPF=9 versus IPF=13; and GSE24206, non-IPF=6 versus IPF=10). Microarray analysis was conducted using R software version 3.02, using the Bioconductor-limma package, and normalized using Robust Multi-array Average (RMA). A Benjamini–Hochberg procedure (false discovery rate) was undertaken to adjust for multiple testing.

### Lysyl oxidase protein expression in lung tissues

Immunohistochemistry was performed using a protocol adapted from Faiz et al. (2013) with antibodies against LOX (Abcam, Cambridge, UK; ab31238; www.abcam.com/lox-antibody-ab31238.html, accessed 2 May 2016), LOXL1 (Novus Biologicals, Littleton, CO, USA; NBP1-82827; www.novusbio.com/LOXL1-Antibody_NBP1-82827.html, accessed 2 May 2016) and LOXL2 (Novus Biologicals; NBP1-32954; www.novusbio.com/Lysyl-Oxidase-Homolog-2-LOXL2-Antibody_NBP1-32954.html, accessed 2 May 2016). Primary antibodies were as follows: LOX (1 µg ml^−1^), LOXL1 (0.1 µg ml^−1^) or LOXL2 (1 µg ml^−1^) and isotype control antibody (same concentration as the respective primary antibodies). Following washing, samples were treated with EnVision+ anti-rabbit horseradish peroxidase (HRP)-conjugated secondary antibody (K4003, Dako, Santa Clara, CA, USA). After a second wash step, colour development was detected with 3′,3′-diaminobenzidine (DAB; Dako), and the tissues were then counterstained in ready-to-use Mayer's Haematoxylin solution (Sigma) for 5 min. Sections were then dehydrated, mounted and coverslipped. All sections for each primary antigen were stained in the same staining run.

### IHC image analysis

Images were captured using a Wide-field FL and TL microscope ZEISS Axio Scan.Z1 Slide Scanner (Zeiss, Oberkochen, Germany). The immunostained tissue was scanned at ×20 magnification, and a whole brightfield tissue image was stitched together from the individual serial images using ZEN Software (Zeiss). All images for each set of immunostaining were captured on the same day with the same microscope settings to ensure uniformity and accuracy of quantified image analysis. Fiji ImageJ (Schindelin et al., 2012) was used to quantify the density and distribution of staining. Colour deconvolution (Ruifrok and Johnston, 2001) vectors in ImageJ were optimized to ensure accurate separation of Eosin and DAB. Overlaid thresholded images of Eosin and DAB were used to measure total tissue surface area. The image analysis calculated the number of pixels above the threshold within the image (‘area’) and the average intensity of the pixels above the threshold (‘average intensity’). Macros were used to batch process the images.

Data were represented as ‘percentage tissue surface area’ (DAB area/total tissue surface area) and ‘density’ (average intensity calculated for the whole tissue).

### Lysyl oxidase protein expression in cell lysates

Western immunoblot was performed using antibodies against LOX (Abcam; ab31238; 1:2000), LOXL1 (Novus Biologicals; NBP1-82827; 1:2000) and LOXL2 (Novus Biologicals; NBP1-32954; 1:2000) using a protocol adapted from Burgess et al. (2003). Cell lysates were run on 10% polyacrylamide gels and transferred to polyvinyledene difluoride membranes (Merck Millipore, Darmstadt, Germany) and protein blocked for 1 h in 5% skimmed milk. After washing, samples were treated with HRP-conjugated secondary antibody (Dako). After a second wash step, detection was performed using chemiluminsecent reagent Immobilon Western (Merck Millipore) and bands were analysed using a Kodak imaging system and Carestream MI software (Molecular Bioimaging, Bend, OR, USA). Membranes were stripped and reprobed for GAPDH (Merck Millipore; 1:5000 dilution) as above as a loading control.

Adjustments of brightness and contrast as indicated in the figure legends were applied uniformly for every set of images and used for display purposes only.

### Antifibrotic potential of LO activity inhibitors (collagen gel 3D model)

Collagen gel solution was prepared for a final collagen gel concentration of 2 mg ml**^−^**^1^. Briefly, 10% (v/v) of 10× PBS and 10% (v/v) 0.1 M NaOH were added to stock rat-tail collagen I (BD Biosciences, Franklin Lakes, NJ, USA) gel solution and mixed while cooled on ice. DMEM media [DMEM low glucose (Gibco) with 0.025 M HEPES (Gibco) and 0.375% sodium hydrogen carbonate (Ajax Finechem)] containing resuspended cells was then added at higher cell concentration for dilution in the collagen gel solution (final concentration of 2.5×10^5^ cells ml^−1^ in the collagen gels). Collagen gel mixed with cells (*n*=5 non-IPF, *n*=5 IPF cell lines and *n*=1 no-cell control) was pipetted into a 48-well plate and allowed to set in a humidified CO_2_ incubator at 37°C for 2 h. Once the gel had set, the gels were then released from the wells by gently running a sterile syringe needle around between the gel and the wall of the wells. Treatment media [Set 1: 0.1% bovine serum albumin (BSA) ±10 ng ml^−1^ TGF-β ±0.1 mM β-APN; Set 2: 0.01% DMSO +0.1% BSA ±10 ng ml^−1^ TGF-β ±300 nM Compound A (Pharmaxis, Sydney, Australia)] of the same volume as the gel were pipetted gently on top of the gels in the wells. The collagen gels were collected at day 7, fixed in 4% paraformaldehyde solution and paraffin embedded.

### Decellularization of human lung tissues

Decellularized tissues from a non-diseased subject and an IPF subject were prepared as previously described (Booth et al., 2012). Essentially, lung tissues were sequentially incubated in sterile water, deoxycholic acid (Sigma), sodium chloride (PanReac) and DNAse (Sigma) to clear cellular and nuclear materials from the tissues. Tissues were washed three times in sterile PBS between each step. The resultant acellular tissue was biopsied using a 12-mm punch and then sliced into 1-mm slices on a vibratome. The acellular tissues slices were sterilized in peracetic acid (Sigma)/ethanol and rinsed extensively in sterile PBS. Slices were stored in sterile PBS at 4°C until use.

### Antifibrotic potential of LO activity inhibitors (decellularized matrix 3D model)

Decellularized lung slices from both non-diseased and IPF subjects were washed three times in sterile DMEM solution and were subsequently added to a suspension of 5.0×10^5^ fibroblasts ml**^−^**^1^ in growth media at a ratio of 1 matrix:2 ml of cell solution (*n*=5 non-IPF, *n*=5 IPF cell lines and *n*=1 no-cell control) up to a maximum of 25 ml of solution in a 50 ml Falcon tube. The Falcon tubes containing the matrices and cell suspension were mounted on a suspension mixer (Selby, ThermoFisher Scientific, Waltham, MA, USA) and constantly rotated at low speed (approximately one rotation every 20 s) at 37°C for 24 h. The reseeded ECMs were placed in separate wells of a 48-well plate with fresh media [0.01% DMSO +0.1% BSA ±10 ng ml**^−^**^1^ TGF-β (R&D Systems, MN, USA) ±300 nM Compound A or 0.1 mM β-APN (Sigma)] and incubated at 37°C and 5% CO_2_. The reseeded decellularized matrices were collected at day 7; half were fixed in 4% paraformaldehyde solution for 24 h and paraffin embedded, whereas the other half were fresh frozen in optimal cutting temperature (OCT) compound and stored at −80°C.

### Tissue stiffness measurements using atomic force microscopy

AFM measurements were performed on decellularized human lung matrices with or without reseeded non-IPF cells. Matrices were embedded in optimal OCT compound, and 10-µm-thick tissue slices were cryosectioned at −21°C and mounted on poly-L-lysine-coated glass slides. PBS solution was added on the tissue slice to avoid drying.

Parenchymal tissue areas were identified under an optical microscope (×200 magnification; Olympus, Tokyo, Japan) and mechanically analysed with a BioScope Catalyst AFM (Bruker, Billerica, MA, USA). Microindentations were performed using a 2.5-µm-radius sphere-tipped probe (Novascan, Ames, IA, USA) with a spring constant determined at ∼100 pN nm^−1^ by the thermal fluctuation method (Thundat et al., 1994). For each sample, three randomly selected areas from three non-consecutive tissue slices were analysed in PBS at room temperature. Force curves were acquired with MIRO 2.0 (NanoScope 9.1; Bruker) at an indentation rate of 20 μm s^−1^ and a ramp size of 10 μm at different points along the parenchymal tissue. More than 100 force curves were performed per sample (∼35 per area; Fig. S8).

The Young's modulus, *E*, was determined by fitting of the force curve by the Hertz sphere model (Dimitriadis et al., 2002; Hertz, 1881) using NanoScope Analysis software (Bruker) and considering Poisson's ratio of 0.4 (Butler et al., 1986).

### Statistical analysis

Data were entered and sorted using Microsoft Excel (Microsoft, Redmond, WA, USA). Sample sizes for comparing IPF versus non-IPF were determined based on a previous study (Tjin et al., 2014). Statistical analysis was performed using GraphPad Prism Version 6 Software (GraphPad, La Jolla, CA, USA). Data were tested for normal distribution with the D'Agostino and Pearson omnibus normality test and analysed with Student's *t*-test (normally distributed) or the Mann–Whitney *U*-test (non-normally distributed) as appropriate. Kruskal–Wallis and two-way ANOVA statistical analyses were performed where appropriate. Factor analysis was performed using SPSS software (IBM, Armonk, NY, USA) using ‘principal axis factoring’ analysing ‘correlation matrix’ and extracting ‘2’ factors from the data set. Promax rotation was used for the factor analysis, using a regression method and excluding cases listwise where there were missing data points.

## Supplementary Material

Supplementary information
