# Correction: Lysyl oxidases regulate fibrillar collagen remodelling in idiopathic pulmonary fibrosis (doi: 10.1242/dmm.030114)

**DOI:** 10.1242/dmm.033191

**Published:** 2017-12-01

**Authors:** Gavin Tjin, Eric S. White, Alen Faiz, Delphine Sicard, Daniel J. Tschumperlin, Annabelle Mahar, Eleanor P. W. Kable, Janette K. Burgess

An error was published in *Dis. Model. Mech.* (2017). **10**, 1301-1312 (doi: 10.1242/dmm.030114).

A reference was incorrectly cited in the Results section (subsection ‘Inhibition of LO activity reduced TGF-β-induced collagen remodelling in collagen I hydrogels’). The corrected sentence and reference are below.

‘To confirm the role of LO enzymes in fibrillar collagen remodelling, 3D *in vitro* cell culture experiments using inhibitors of LO activity were performed. β-APN is a non-selective inhibitor of LO activity, whereas Compound A is a specific inhibitor of LOXL2 activity, referred to as PXS-S2A in a recent publication by Chang et al. (2017).’

Chang, J., Lucas, M. C., Leonte, L. E., Garcia-Montolio, M., Singh, L. B., Findlay, A. D., Deodhar, M., Foot, J. S., Jarolimek, W., Timpson, P. et al. (2017). Pre-clinical evaluation of small molecule LOXL2 inhibitors in breast cancer. *Oncotarget* **8**, 26066-26078. http://doi.org/10.18632/oncotarget.15257.

The authors apologise to readers for this error.

