# Supplementary material for: Lysyl oxidases regulate fibrillar collagen remodelling in idiopathic pulmonary fibrosis
Source: Dis Model Mech. 2017 Nov 1;10(11):1301–12. doi: 10.1242/dmm.030114 (PMC5719253; doi:10.1242/dmm.030114)
Supplement: Supplementary information [file dmm-10-030114-s1.pdf]

Supplementary Tables:

Table S1. LOXL1 Density is increased in IPF compared to non-IPF ILD.

Image Analysis		Non-IPF ILD		IPF		p-value	
		**Mean**	**± s.d.**	**Mean**	**± s.d.**		
**LOX**	**% Surface Area**	86.28	5.42	84.06	6.58		0.396
	**Density**	154.10	15.61	152.20	17.35		0.795
**LOXL 1**	**% Surface Area**	67.53	12.81	72.40	7.65		0.192
	**Density**	168.60	11.47	179.50	11.99	↑	**0.031***
**LOXL 2**	**% Surface Area**	53.65	18.29	44.93	18.00		0.331$^{\delta}$
	**Density**	156.60	15.30	148.30	12.58		0.254$^{\delta}$

Quantification of immunohistochemical staining of the lung tissues using image analysis on whole tissue, data presented as % of Surface Area stained with the protein of interest and Density of staining within the stained surface areas. Comparing non-IPF ILD (N = 8) and IPF (N = 24 - 26) subjects (average from 1 – 2 samples/subject). Data obtained from 1 experimental replicate. Data analysed with Student's 2-tailed parametric unpaired t-test. Abbreviations: ILD = Interstitial Lung Disease, LOX = Lysyl oxidase, LOXL1 = Lysyl oxidase like-1, LOXL2 = Lysyl oxidase like-2, SD = Standard Deviation, ↑ = Significantly increased compared to non-IPF and * = p-value < 0.05, $^{\delta}$ = Mann-Whitney Test.

Table S2. Strength of the contributions of the variables within each relationship group.

Variables	Relationship Groups	
	**1**	**2**
**Area F/B**	0.837	
**Intensity F/B**	0.833	
**LOX % Surface Area**		1.018
**LOX Density**		0.930
**LOXL1 % Surface Area**	1.088	
**LOXL1 Density**	0.999	
**LOXL2 % Surface Area**	0.666	
**LOXL2 Density**	0.517	-0.583

Contribution of collagen maturity/organization as measured by SHG (F/B values) and IHC quantification of LOX, LOXL1 and LOXL2 in human lung. Combined value from non-IPF (N = 7) and IPF (N = 8). Data obtained from experimental 1 replicate. Abbreviations: IPF = Idiopathic Pulmonary Fibrosis, IHC = Immunohistochemistry, LOX = Lysyl oxidase, LOXL1 = LOX like-1, LOXL2 = LOX like-2, SHG = Second Harmonic Generation, F/B = SHG Forward/Backward Signal Ratio.

Table S3. Patient Demographics

Sample #	Age	Sex	Diagnosis	Sample Type	SHG	LOX IHC	LOXL1 IHC	LOXL2 IHC	Western Blot	COL1 Gel	RS DCM
**1**	30	M	Donor	FFPE	X	X	X	X			
**2**	25	M	Donor	FFPE	X	X	X	X			
**3**	22	M	Donor	FFPE	X	X	X	X			
**4**			Donor	FFPE							
**5**	16	M	Donor	FFPE	X	X	X	X			
**6**	52	M	Donor	FFPE	X	X	X	X			
**7**	45	F	Donor	FFPE	X	X	X	X			
**8**	58	M	Donor	FFPE	X	X	X	X			
**9**	64	M	IPF	FFPE	X	X	X	X			
**10**	55	M	IPF	FFPE	X	X	X	X			
**11**	58	M	IPF	FFPE	X	X	X	X			
**12**	63	M	IPF	FFPE, Fibroblasts	X	X	X	X	X	X	X
**13**	56	M	IPF	FFPE	X	X	X	X			
**14**			IPF	FFPE	X	X	X	X			
**15**	57	M	IPF	FFPE	X	X	X	X			
**16**	58	M	IPF	FFPE, Fibroblasts	X	X	X	X	X	X	
**17**	47	M	Donor	FFPE		X	X	X			
**18**			Donor	FFPE		X	X	X			
**19**	20	M	Donor	FFPE		X	X	X			
**20**			IPF	FFPE	X	X	X	X			
**21**			IPF	FFPE	X		X	X			
**22**			IPF	FFPE	X	X	X	X			
**23**			IPF	FFPE	X	X	X	X			
**24**			IPF	FFPE	X	X	X	X			
**25**			IPF	FFPE	X	X	X	X			

**26**			IPF	FFPE	X	X	X	X			
**27**			IPF	FFPE	X	X	X	X			
**28**			IPF	FFPE	X	X	X	X			
**29**			IPF	FFPE	X	X	X	X			
**30**			IPF	FFPE	X	X	X	X			
**31**			IPF	FFPE	X	X	X	X			
**32**			IPF	FFPE	X	X	X	X			
**33**			IPF	FFPE							
**34**			IPF	FFPE	X	X	X	X			
**35**			IPF	FFPE	X	X	X	X			
**36**			IPF	FFPE	X	X	X	X			
**37**			IPF	FFPE	X	X	X	X			
**38**			IPF	FFPE	X	X	X	X			
**39**			IPF	FFPE	X	X	X	X			
**40**			IPF	FFPE	X		X				
**41**			IPF	FFPE	X	X	X	X			
**42**			IPF	FFPE	X	X	X	X			
**43**			IPF	FFPE	X	X	X	X			
**44**			IPF	FFPE	X	X	X	X			
**45**			IPF	FFPE	X	X	X	X			
**46**			IPF	FFPE	X	X	X	X			
**47**			Non-IPF ILD	FFPE	X	X	X	X			
**48**			Non-IPF ILD	FFPE	X	X	X	X			
**49**			Non-IPF ILD	FFPE	X	X	X	X			
**50**			Non-IPF ILD	FFPE	X	X	X	X			
**51**			Non-IPF ILD	FFPE	X	X	X	X			
**52**			Non-IPF ILD	FFPE	X	X	X	X			
**53**			Non-IPF ILD	FFPE	X	X	X	X			
**54**			Non-IPF ILD	FFPE	X	X	X	X			
**55**	73	F	NSCCa	Fibroblasts					X	X	X
**56**	72	M	NSCCa	Fibroblasts					X	X	X
**57**	72	M	NSCCa	Fibroblasts					X	X	X

Disease Models & Mechanisms • Supplementary information

**58**	54	M	NSCCa	Fibroblasts						X	X
**59**	61	M	NSCCa	Fibroblasts					X	X	X
**60**	54	M	IPF	Fibroblasts					X	X	X
**61**	65	M	IPF	Fibroblasts					X	X	X
**62**	50	M	IPF	Fibroblasts					X	X	X

Abbreviations used: M = Male, F = Female, IPF = Idiopathic Pulmonary Fibrosis, ILD = interstitial lung disease, NSCCa = Non-Small Cell Carcinoma, FFPE = Formalin Fixed-Paraffin Embedded, SHG = Second Harmonic Generation, LOX = Lysyl oxidase, LOXL1 = LOX like-1, LOXL2 = LOX like-2, COL1 Gel = Collagen I Hydrogel, RS DCM = Reseeded Decellularised Matrices.

Supplementary Figures:

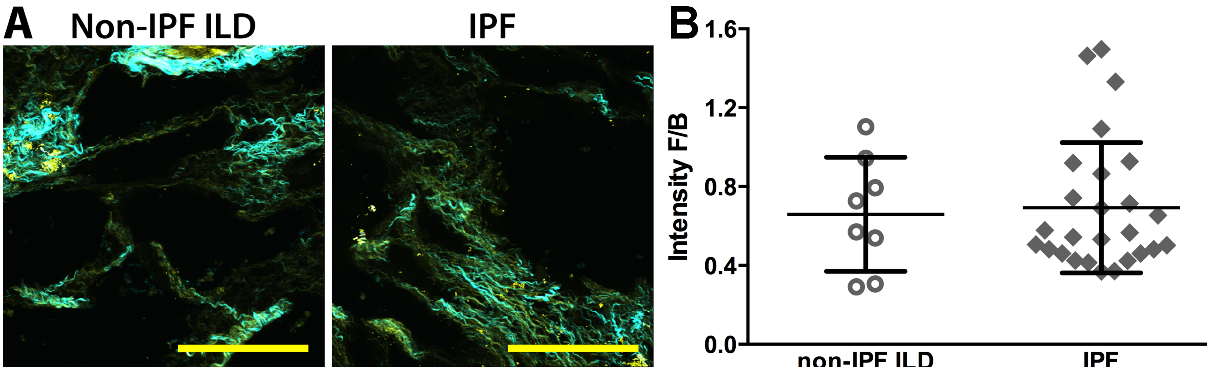

Fig. S1. Collagen fibrillar remodelling in IPF is a feature of pulmonary fibrosis and not specific to the disease. (A and B) Second Harmonic Generation (SHG) Imaging and image analysis of human lung parenchyma. (A) Representative SHG images of lung parenchyma from Non-IPF ILD (N = 8; open circles) and IPF (N = 26; filled diamonds) subjects (Yellow = Backward Immature/Disorganized Collagen; Cyan = Mature/Organized Collagen; scale bar = 100 μm). Brightness and contrast has been enhanced for display purposes only and applied equally to all images. (B) Image analysis quantification of SHG Intensity Forward/Backward Signal Ratio (Intensity F/B), higher ratio indicates higher proportion of organized/mature fibrillar collagen. Data analyzed with Student's 2-tailed parametric unpaired t-test. Data presented as mean ± s.d. Data obtained from 1 experimental replicate. Abbreviations ILD = Interstitial Lung Disease.

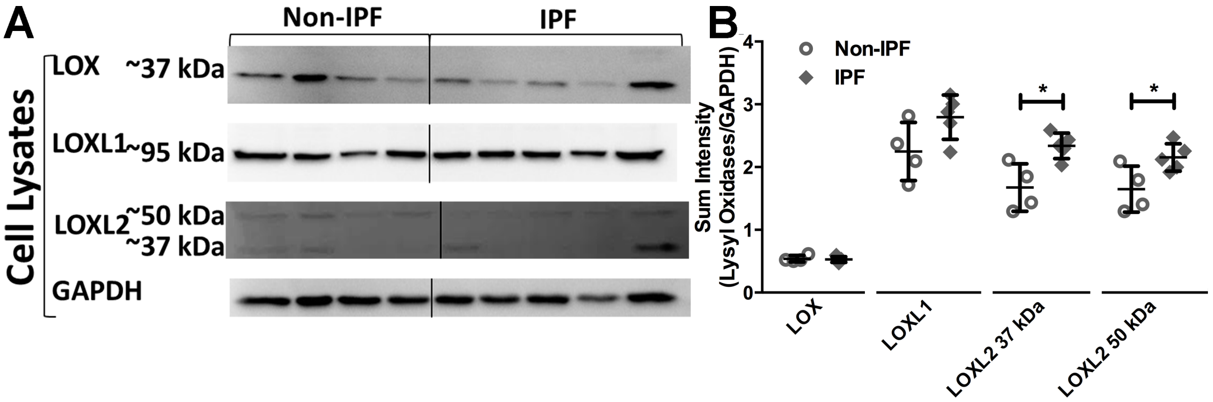

Fig. S2. Lysyl oxidase enzymes are differentially expressed in IPF cells. (A) Western blot images of cell lysates from non-IPF (n=4) and IPF (n=5) primary human lung fibroblasts probed for LOX, LOXL1, LOXL2 and GAPDH. Brightness and contrast has been enhanced for display purposes only. (B) Densitometric analysis of Western blot, data presented as intensity of Lysyl oxidases normalized to GAPDH as loading control for cell lysates (open circles = non-IPF, filled diamonds = IPF). Data analysed with Student's 2-tailed parametric unpaired t-test. Data presented as mean ± s.d. Data obtained from 1 experimental replicate. IPF = Idiopathic Pulmonary Fibrosis, LOX = Lysyl oxidase, LOXL1 = LOX like-1, LOXL2 = LOX like-2 and GAPDH = Glyceraldehyde 3-phosphate dehydrogenase, * = p > 0.05.

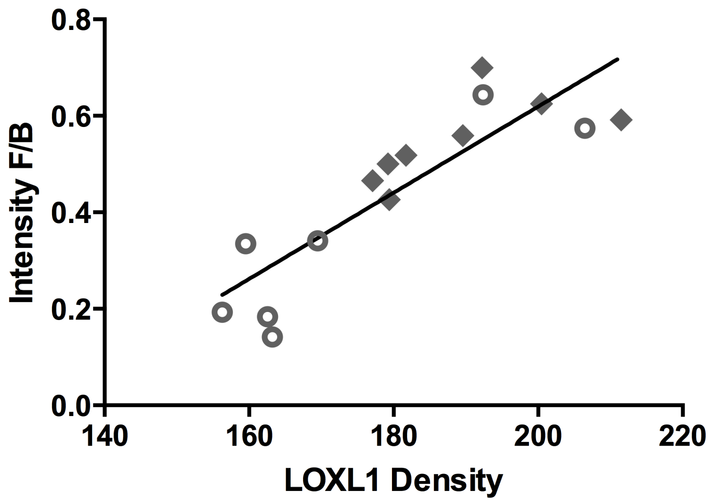

Fig. S3. LOXL1 enzyme expression correlates with the maturity/organization of the fibrillar collagen in lung tissues. Image analysis quantification of SHG Intensity Forward/Backward Signal Ratio (Intensity F/B) correlated with quantification of LOXL1 immunohistochemical staining of the lung tissues using image analysis on whole tissue (data presented as density of staining) from non-IPF (N = 7; open circles) and IPF (N = 8; filled diamonds) subjects (p-value = <0.0001; R^2-value = 0.7506) (average of 3 – 4 samples/subject). Data obtained from 1 experimental replicate. Abbreviations: IPF = Idiopathic Pulmonary Fibrosis and LOXL1 = Lysyl oxidase like-1.

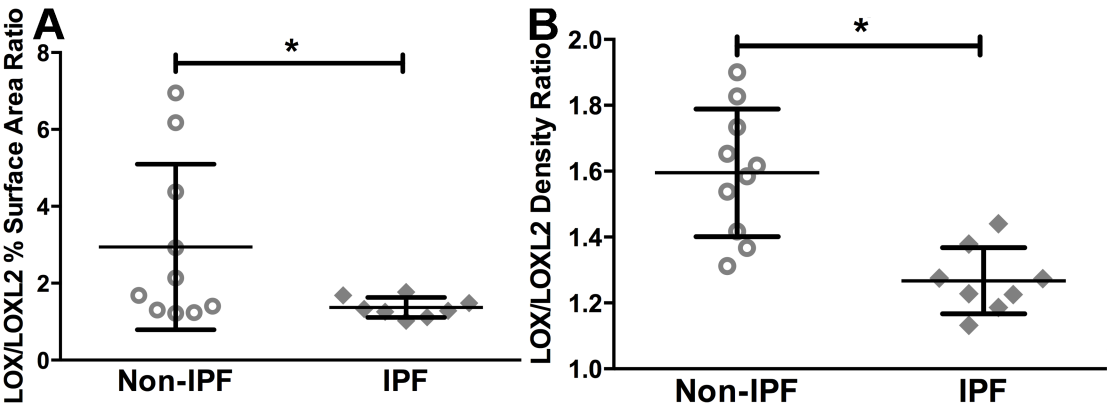

Fig. S4. LOX and LOXL2 ratio is significantly decreased in IPF. The ratio of LOX and LOXL2 (A) % Surface Area and (B) Density between non-IPF (N = 10; open circles) and IPF (N = 8; filled diamonds) subjects. *** = $p < 0.005$. Data obtained from 1 experimental replicate. Data analyzed with Student's 2-tailed parametric unpaired t-test. Data presented as mean ± s.d. Abbreviations: IPF = Idiopathic Pulmonary Fibrosis, LOX = Lysyl oxidase and LOXL2 = Lysyl oxidase like-2.

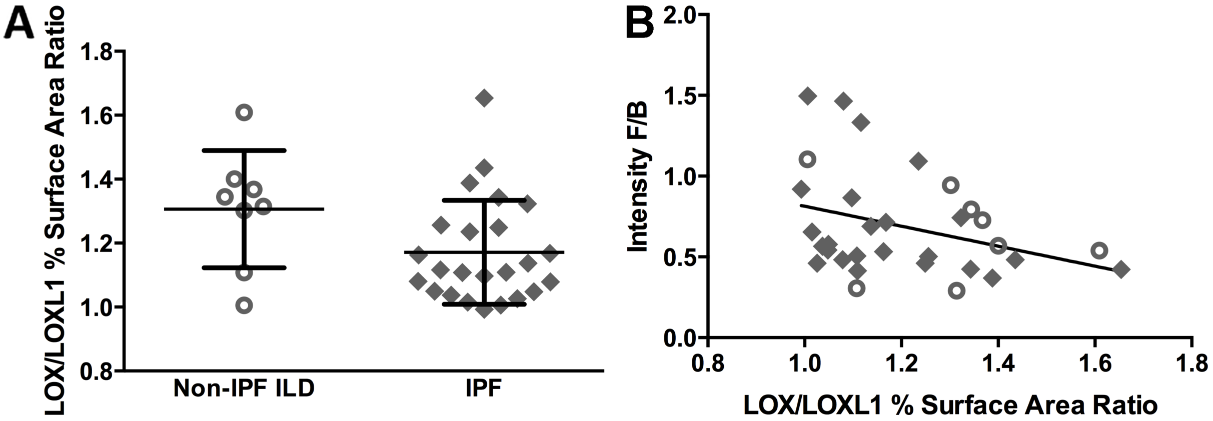

Fig. S5. The differential expression of LOX family enzymes may be specific for IPF. (A) The ratio of LOX and LOXL1 % Surface Area between non-IPF ILD (N = 8; open circles) and IPF (n = 24; filled diamonds) subjects; Data analyzed with Student's 2-tailed parametric unpaired t-test. Data presented as mean ± s.d. (B) The correlation of the ratio of LOX and LOXL1 with F/B ratio in non-ILD and IPF subjects (p-value = 0.0575; R^2-value = 0.1151). Abbreviations: IPF = Idiopathic Pulmonary Fibrosis, LOX = Lysyl oxidase and LOXL1 = Lysyl oxidase like-1. Data obtained from experimental 1 replicate.

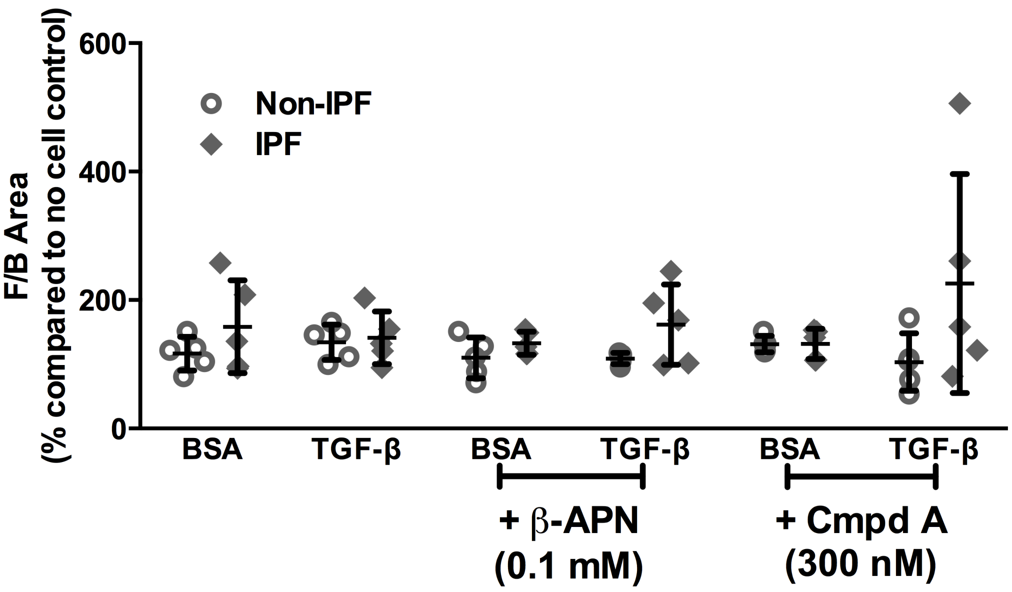

Fig. S6. Inhibition of lysyl oxidase activity did not affect amount of mature collagen fibres. Decellularised non-IPF matrices re-seeded with primary human lung parenchymal fibroblasts (10^6 cells/matrix) were stimulated with TGF-β (10 ng ml^{-1}) in the presence or absence of the pan-lysyl oxidase inhibitor (β-APN) (100 μM) or the LOXL2 specific inhibitor (Compound A) (300 nM). Cells were seeded in the matrix for 24 hours before being transferred into separate tissue culture wells with treatment media for 7 days. Samples were collected at the end of the treatment period and formalin fixed and paraffin embedded. Quantification of SHG microscopy on decellularised non-IPF matrices re-seeded for 7 days with fibroblasts from control (n = 5; open circles) and idiopathic pulmonary fibrosis (IPF) (n = 5; filled diamonds) subjects treated with the pan-lysyl oxidase inhibitor (β-APN) (100 μM) or the LOXL2 specific inhibitor (Compound A) (300 nM) in the presence or absence of TGF-β (10 ng ml-1) (data presented as % of respective no cell control, Mean ± s.d.); SHG Forward/Backward Signal Area Ratio (F/B Area), higher ratio indicates greater amount of mature fibrillar collagen fibres. Statistical Analysis used was Two-way ANOVA with matching and multiple comparisons with Tukey's correction. Data presented as mean ± s.d. Data obtained from 1 replicate.

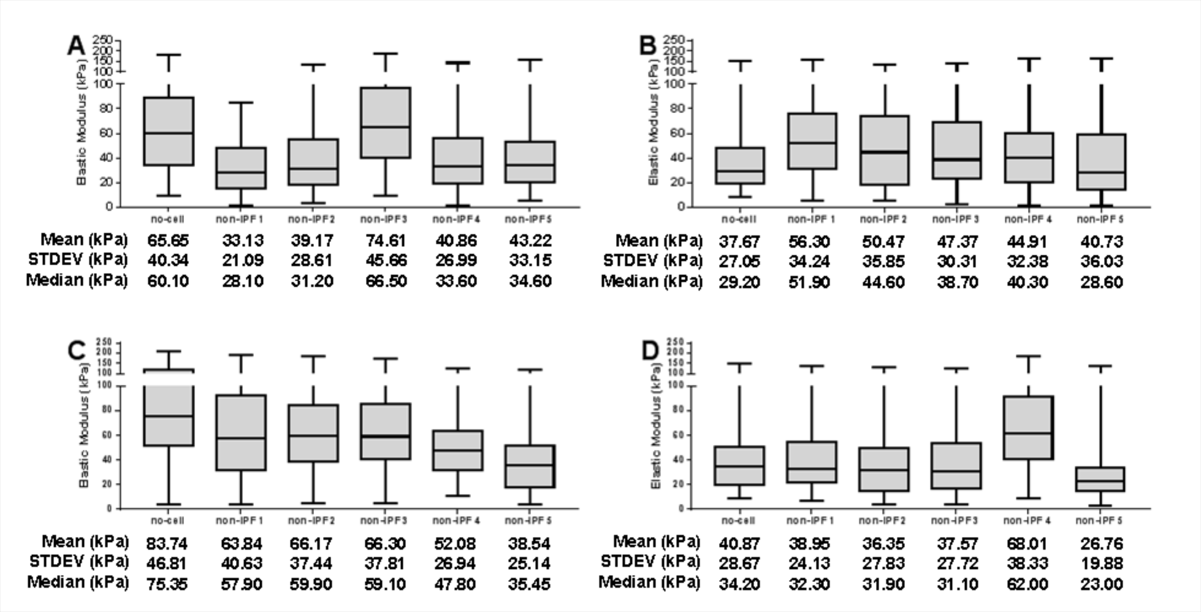

Fig. S7. AFM measurements of each treatment for each sample. Decellularised non-IPF matrices re-seeded with primary human lung parenchymal fibroblasts (10^6 cells/matrix) were stimulated with TGF-β (10 ng ml^{-1}) in the presence or absence of the pan-lysyl oxidase inhibitor (β-APN) (100 µM). Cells were seeded in the matrix for 24 hours before being transferred into separate tissue culture wells with treatment media for 7 days. Samples were collected at the end of the treatment period and fresh frozen in OCT compound (10 µm slices). Box and whiskers plot of the stiffness (elastic modulus) for (A) BSA, (B) BSA + β-APN, (C) TGF-β and (D) TGF-β + β-APN. Stiffness was measured with AFM and >100 measurements spread over 3 areas were taken per sample. Each n is a single AFM measurement. Data is obtained from 1 experimental replicate. Abbreviations: AFM = Atomic force microscopy, β-APN = β-aminoproprionitrile, BSA = Bovine serum albumin, TGF-β = Transforming growth factor-β, OCT = Optimal cutting temperature ** = p<0.01, **** = p<0.0001.

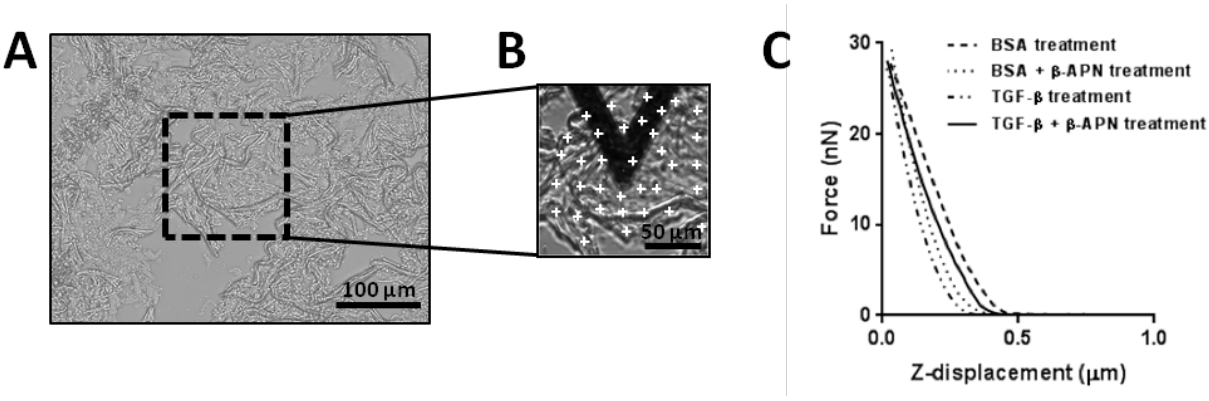

Fig. S8. Setup of atomic force microscopy measurement. (A) Representative bright field image (x200) of human lung decellularized matrice re-seeded with non-IPF cells. Scale bar = 100 µm. (B) Optical image of AFM tip (triangular cantilever) indenting lung tissue. White crosses indicate the location of indentation measurement (35 points per area) on the parenchyma tissue. Scale bar = 50 µm. (C) Representative force curves performed on samples treated with BSA and TGF-β.

References

Abraham, T., Kayra, D., McManus, B. and Scott, A. (2012). Quantitative assessment of forward and backward second harmonic three dimensional images of collagen Type I matrix remodeling in a stimulated cellular environment. *J Struct Biol* **180**, 17-25.

Booth, A. J., Hadley, R., Cornett, A. M., Dreffs, A. A., Matthes, S. A., Tsui, J. L., Weiss, K., Horowitz, J. C., Fiore, V. F., Barker, T. H. et al. (2012). Acellular normal and fibrotic human lung matrices as a culture system for in vitro investigation. *Am J Respir Crit Care Med* **186**, 866-76.

Faiz, A., Tjin, G., Harkness, L., Weckmann, M., Bao, S., Black, J. L., Oliver, B. G. and Burgess, J. K. (2013). The expression and activity of cathepsins D, H and K in asthmatic airways. *PLoS One* **8**, e57245.

Ruifrok, A. C. and Johnston, D. A. (2001). Quantification of histochemical staining by color deconvolution. *Anal Quant Cytol Histol* **23**, 291-9.

Schindelin, J., Arganda-Carreras, I., Frise, E., Kaynig, V., Longair, M., Pietzsch, T., Preibisch, S., Rueden, C., Saalfeld, S., Schmid, B. et al. (2012). Fiji: an open-source platform for biological-image analysis. *Nat Methods* **9**, 676-82.

Tjin, G., Xu, P., Kable, S. H., Kable, E. P. and Burgess, J. K. (2014). Quantification of collagen I in airway tissues using second harmonic generation. *J Biomed Opt* **19**, 36005.

Disease Models & Mechanisms • Supplementary information
